# Hyperbaric oxygen attenuates neuropathic pain and reverses inflammatory signaling likely via the Kindlin-1/Wnt-10a signaling pathway in the chronic pain injury model in rats

**DOI:** 10.1186/s10194-016-0713-y

**Published:** 2017-01-05

**Authors:** Baisong Zhao, Yongying Pan, Haiping Xu, Xingrong Song

**Affiliations:** Department of Anesthesiology, Guangzhou Women and Children’s Medical Center, Guangzhou Medical University, No. 9 Jinsui Road, Tianhe District, Guangzhou, Guangdong 510623 China

**Keywords:** Hyperbaric oxygen, Neuropathic pain, Chronic constriction injury, Kindlin-1, Wnt-10a, Astrocyte activation

## Abstract

**Background:**

Hyperbaric oxygen (HBO) therapy is proven to attenuate neuropathic pain in rodents. The goal of the present study was to determine the potential involvement of the Kindlin-1/Wnt-10a signaling pathway during astrocyte activation and inflammation in a rodent model of neuropathic pain.

**Methods:**

Rats were assigned into sham operation, chronic constriction injury (CCI), and CCI + HBO treatment groups. Neuropathic pain developed in rats following CCI of the sciatic nerve. Rats in the CCI + HBO group received HBO treatment for five consecutive days beginning on postoperative day 1. The mechanical withdrawal threshold (MWT) and the thermal withdrawal latency (TWL) tests were performed to determine mechanical and heat hypersensitivity of animals, respectively. Kindlin-1, Wnt-10a and β-catenin protein expression was examined by immunohistochemistry and Western blot analysis. Expression of tumor necrosis factor (TNF)-α was also determined by ELISA.

**Results:**

Our findings demonstrated that HBO treatment significantly suppressed mechanical and thermal hypersensitivity in the CCI neuropathic pain model in rats. HBO therapy significantly reversed the up-regulation of Kindlin-1 in dorsal root ganglia (DRG), spinal cord, and hippocampus of CCI rats. CCI-induced astrocyte activation and increased levels of TNF-α were efficiently reversed by HBO (*P* < 0.05 *vs.* CCI). HBO also reversed Wnt-10a up-regulation induced by CCI in the DRG, spinal cord, and hippocampus (*P* < 0.05 *vs.* CCI).

**Conclusions:**

Our findings demonstrate that HBO attenuated CCI-induced rat neuropathic pain and inflammatory responses, possibly through regulation of the Kindlin-1/Wnt-10a signaling pathway.

## Background

Neuropathic pain, characterized by allodynia, spontaneous pain, hyperalgesia, and paraesthesia, is among the most difficult types of chronic pain to clinically treat arising from its complex etiology [1]. A large body of evidence has shown that the quality of life for patients with neuropathic pain is poor [2, 3]. Multiple concurrent mechanisms are implicated in the processing of pain signals, and thus, administration of combinations of drugs has been recommended for the management of this debilitating type of pain. However, clinical efficacy and side effects of using combination pharmacotherapy remains unclear and variable [4]. Hence, novel strategies targeting neuropathic pain are much needed clinically. Indeed, non-pharmacological approaches, such as transcutaneous electrical nerve stimulation [5], transcranial magnetic stimulation [6], and electro acupuncture [7] have been proven to significantly alleviate neuropathic pain. Nevertheless, the long-term efficacy of these approaches is yet to be determined.

Hyperbaric oxygen (HBO) is a form of medical treatment in which patients breath 100% oxygen under increased atmospheric pressure [8]. Emerging lines of evidence suggest that HBO therapy can reduce chronic pain in animal models [9–11]. In addition, the use of HBO treatment has been proven to attenuate chronic cluster headaches and [12] idiopathic trigeminal neuralgia in patients [13]. Our previous study indicated that the antinociceptive effects of HBO therapy may be linked to inhibition of astrocyte activation and inflammation in a chronic constriction injury (CCI)-induced neuropathic pain model in rats [10]. However, the precise molecular mechanisms underpinning this process are currently unknown.

Although the pathogenesis of neuropathic pain has not yet been fully described, chronic inflammation caused by astrocyte activation is believed to be one of the most crucial events in the development of this condition [14]. The activation of astrocytes in response to stimuli is driven by β1 integrins [15]. Kindlin-1 is an integrin binding protein which plays a key role in regulating integrin activity [16]. A recent study demonstrated that Kindlin-1 controls cell proliferation through regulation of the Wnt/β-catenin signaling pathway [17]. Nevertheless, the potential involvement of the Kindlin-1/Wnt signaling pathway in the observed antinociceptive effects yielded by HBO treatment is still unclear.

Here, we analyzed the neuropathic pain relieving effects of HBO treatment by using a rodent CCI-induced pain model. In addition, the potential activation of Kindlin-1/Wnt signaling during astrocyte activation and inflammatory responses upon neuropathic pain induction was determined. Our results showed that HBO therapy attenuated neuropathic pain in rats, possibly through regulating Kindlin-1/Wnt-10a signaling, astrocyte activation, and subsequent inflammatory responses. These findings provide a basic understanding of the molecular mechanism of neuropathic pain pathogenesis, and suggest that HBO therapy may be a promising non-pharmacological strategy for the management of neuropathic pain.

## Methods

### Animals and experimental groups

Thirty-six 8–10 week-old male Sprague-Dawley (SD) rats, weighing 250–280 g, were obtained from the Guangdong Medical Laboratory Animal Center, Foshan, Guangdong, China (Animal license No. SCXK 2013–0002). The animals were housed individually in plastic boxes at a controlled room temperature of 23–25 °C. Animals had free access to water and food. Thirty-six rats were randomly assigned into three groups with 12 animals per group, including sham operation control group, CCI group, and CCI + HBO group. All efforts were made to minimize suffering of the animals. The animal study was carried out in strict accordance with the recommendations in the Guide for the Care and Use of Laboratory Animals of the National Institutes of Health, and under approved protocols of the Institutional Animal Ethics Committee of the Guangzhou Medical University (2016–016).

### Establishment of the chronic constriction injury (CCI) neuropathic pain model in rats

CCI of the sciatic nerve was applied to induce neuropathic pain in rats [10, 11]. In brief, animals were given 40 mg/kg body weight of sodium pentobarbital by intraperitoneal injection. Under anesthesia, the left sciatic nerve of the rats in the CCI or the CCI + HBO group was exposed at the mid-thigh level following blunt dissection of the left biceps femoris. Four ligatures were then loosely tied around the exposed sciatic nerve at the proximal area of the trifurcation at 1 mm intervals. After induction of CCI, the wound was closed. An identical dissection was carried out in animals from the sham operation group, however, the sciatic nerve in these control rats was not ligated.

### Hyperbaric oxygen (HBO) treatment

A cylindrical HBO chamber (DS400-IV; Weifang Huaxin Oxygen Industry Co., Ltd., Shandong, China) was utilized for HBO treatment as described previously [10, 11]. Briefly, the chamber was filled with 100% oxygen continuously before experiments. Animals in the CCI + HBO group were then placed into the chamber. The pressure within the chamber was increased at a rate of 0.1 ATA/min to 2.0 ATA/min within 40 min, and was maintained for one hour. At the end of the therapy, the pressure was gradually decompressed to atmospheric pressure at a rate of 0.1 ATA/min. Animals in the CCI + HBO group received HBO treatment for five consecutive days starting from postoperative day 1. Animals in the sham operation control or CCI groups were allowed to stay in the chamber for 100 min, but without any treatment.

### Behavioral analysis

Behavioral tests were conducted on eight successive days starting from the day prior to the operation. Rats were habituated in a plexiglas chamber (Youer Equipment Scientific Co., Ltd., Shanghai, China) for 1 h before testing.

The mechanical withdrawal threshold (MWT) test was performed to examine the paw response to mechanical stimuli as previously described [11]. During the MWT test, animals were placed in the chamber after habituation, and von Frey filaments (Stoelting Company, Wood Dale, IL, USA) were used to stimulate the left hind paw of each rat. The stimulation duration was approximately 3–5 s, with an interval of 30 s. A 0.6 g von Frey force was directed on the plantar surface of the hind paw following the up-and-down force stimulation as described by Song et al. [18]. A positive response was defined as an immediate withdrawal of the hind paw in response to stimulation. A reduced force was generated if the foot withdrawal occurred. In contrast, an increased force was induced when a negative response occurred. Such procedure was repeated until we identified the least force that evoked withdrawal. The withdrawal threshold was set as the von Frey force that induced 50% paw withdrawal. For each animal, the MWT test was conducted ten times. A cut-off value of the von Frey force was set at 15 g.

In order to examine paw sensitivity in response to thermal stimulus, the thermal withdrawal latency (TWL) assay was applied as reported previously [11]. Upon testing, animals were placed on the surface of a glass plate (3 mm thick) covered by a Plexiglas chamber with a fully-automatic plantar analgesia tester (BME-410C) obtained from Youer Equipment S cientific Co., Ltd., Shanghai, China. The left hind paw of the animal was exposed to a heat stimulus. The duration of paw withdrawal from the heat source was considered as TWL. For each trial, five thermal stimuli at five min intervals were delivered, and the average TWL was calculated. A cut-off value of 30 s was set.

### Sample preparation

After completing behavioral experiments 7 days post-operatively, all rats were given 40 mg/kg sodium pentobarbital intraperitoneally to induce anesthesia. Under anesthesia, animals were transcardially perfused with saline. Subsequently, the L5 dorsal root ganglion (DRG), spinal cord areas between L4 and L6 segments as well as the hippocampus were carefully removed and used for immunohistochemical analysis (four rats for each group). Other tissues were used for Western blotting experiment (four rats per group) and ELISA (four rats per group).

### Immunohistochemical analysis

DRG, spinal cord, and hippocampal tissue sections (5 μm thick) were incubated at 4 °C overnight with a mouse anti-rat anti-Kindlin-1 (1:500 dilution, MAB2616; Millipore, Billerica, MA, USA), rabbit anti-rat anti-Wnt-10a (1:500 dilution, ab106522; Abcam, Cambridge, UK), rabbit anti-rat anti-glial fibrillary acidic protein (GFAP) (1:200 dilution, ab7260; Abcam, USA) antibody followed by incubation with biotinylated secondary antibody (1:200 dilution; Vector Laboratories, Burlingame, CA, USA) in 1.5% normal donkey serum (NDS; Jackson Immuno Research Laboratories Inc., West Grove, PA, USA) for 20 min at 37 °C. Nuclei were stained with DAPI (4′,6-diamidino-2-phenylindole). All sections were examined under a confocal laser scanning microscope (Leica SP2, Wetzlar, Germany). For each animal, DRG, spinal cord and hippocampal sections (eight for each sample) were randomly selected for data quantification. The intensity of the optical density (OD) was calculated using Image J analysis software (National Institutes of Health, Bethesda, MD, USA) from eight sections for each animal. For each section, five fields were randomly selected under microscope. The percentage of GFAP-positive astrocytes cells (activated astrocytes) was calculated over the total number of cells. The average percentage of activated astrocytes was calculated. Data quantification of immunohistochemical analysis was carried out blindly with respect to treatments.

### Western blot analysis

Total protein was extracted from tissues using SDS lysis buffer (P0013G; Beyotime, China) for evaluating the protein expressions of Kindlin-1, Wnt 10a, and β-catenin. The concentration of extracted protein samples was examined using the Pierce BCA assay. Equal amounts of protein were separated by sodium dodecyl sulfate-polyacrylamide gel electrophoresis (SDS-PAGE). Clectrophoresis protein samples were then transferred to a polyvinylidene fluoride membrane. After blocking in phosphate buffer solution (PBS) containing 5% w/v non-fat milk at 37 °C for 1–2 h, the membranes were incubated with rabbit monoclonal anti-Kindlin-1 primary antibody (1:1000 dilution; Millipore, USA), anti-Wnt-10a antibody (1:500 dilution, Abcam, USA), or anti-β-catenin antibody (1:1000 dilution, Abcam, USA) overnight at 4 °C. Antibodies against β-actin were used as an internal control. After washing with PBS-T (0.1% Tween-20 in PBS) four times, membranes were treated with horseradish peroxidase (HRP)-labeled secondary antibodies (1:2000 dilution; Santa Cruz, Santa Cruz, CA, USA) at 37 °C for 1 h. Immunolabeled protein bands were visualized using an enhanced chemiluminescence (ECL) assay (E-CS-0100c, Elabscience, Wuhan. China). The bands of target proteins were analyzed using the Scion Image software version 4.0.3 (Scion Corp., Frederick, MD, USA). The densitometric values were used for statistical analysis.

### Determination of tumor necrosis factor (TNF)-α levels

The concentration of TNF-α in the hippocampus was measured by ELISA as described previous [19]. The unilateral hippocampus, DRG, or spinal cord was dissected, ground with a grinder, and loaded onto an ultrasonic tissue homogenizer. The supernatant was collected after centrifugation. TNF-α production was evaluated using ELISA kits (Boster Biological Technology Co., Ltd., Wuhan, China). OD values at 490 nm were recorded using a microplate reader (NK3; Ladsystems, Helsinki, Finland). The average level of TNF-α was calculated based on a standard curve provided by the kit.

### Statistical analysis

Data were analyzed by the SPSS 17.0 software (SPSS Inc., Chicago, IL, USA). Numerical data are presented as the mean ± standard deviation. In order to compare the differences among groups, one-way analysis of variance (ANOVA), followed by the least significant difference test, was applied. *P* < 0.05 was considered statistically significant.

## Results

### HBO reduced mechanical and thermal hypersensitivity in rodents with neuropathic pain

**Fig. 1:**
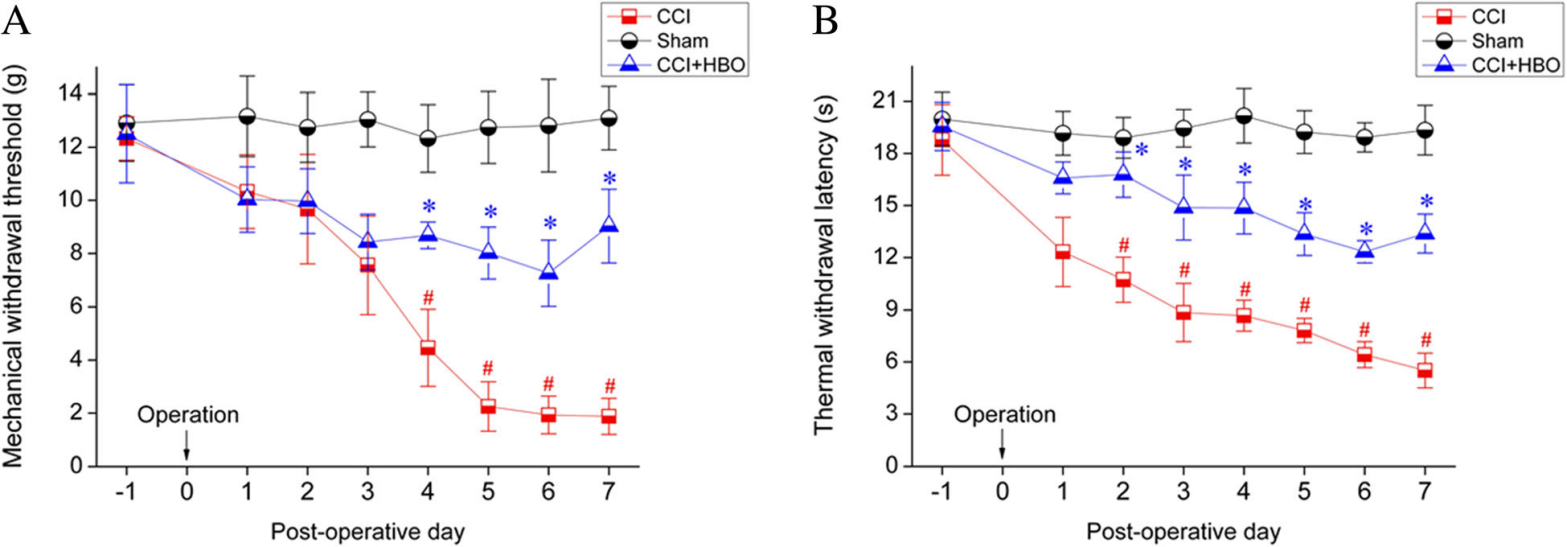
Mechanical and thermal hypersensitivity in rats. Animals in sham operation control (*n* = 12), CCI (*n* = 12), and CCI + HBO (*n* = 12) groups were subjected to behavioral tests on preoperative day 1 and 7 after operation. Mechanical and thermal hypersensitivity in animals was determined by measuring the mechanical withdrawal threshold (MWT) (**a**) and the thermal withdrawal latency (TWL) (**b**). ^#^ *P* < 0.05 *vs.* sham; *P < 0.05 *vs.* CCI

To induce neuropathic pain in rats, CCI of the sciatic nerve was applied. MWT and TWL levels gradually decreased with time after surgery in animals of the CCI group when compared with sham operation control rats (Fig. 1). Development of significant mechanical hypersensitivity was observed in CCI rats after postoperative day 4 (*P* < 0.05 *vs.* sham) and heat hypersensitivity developed after postoperative day 2 (*P* < 0.05 *vs.* sham). These data suggest the successful establishment of a neuropathic pain model in rats.

HBO treatment greatly prevented the reduction in MWT and TWL values induced by CCI (MWT, *P* < 0.05 *vs.* CCI after postoperative day 4; TWL, *P* < 0.05 *vs.* CCI after postoperative day 2). These findings indicate that five consecutive days of HBO treatment by suppressing mechanical and thermal hypersensitivity efficiently relieved neuropathic pain in rats induced by CCI.

### HBO reversed Kindlin-1 up-regulation induced by CCI

**Fig. 2:**
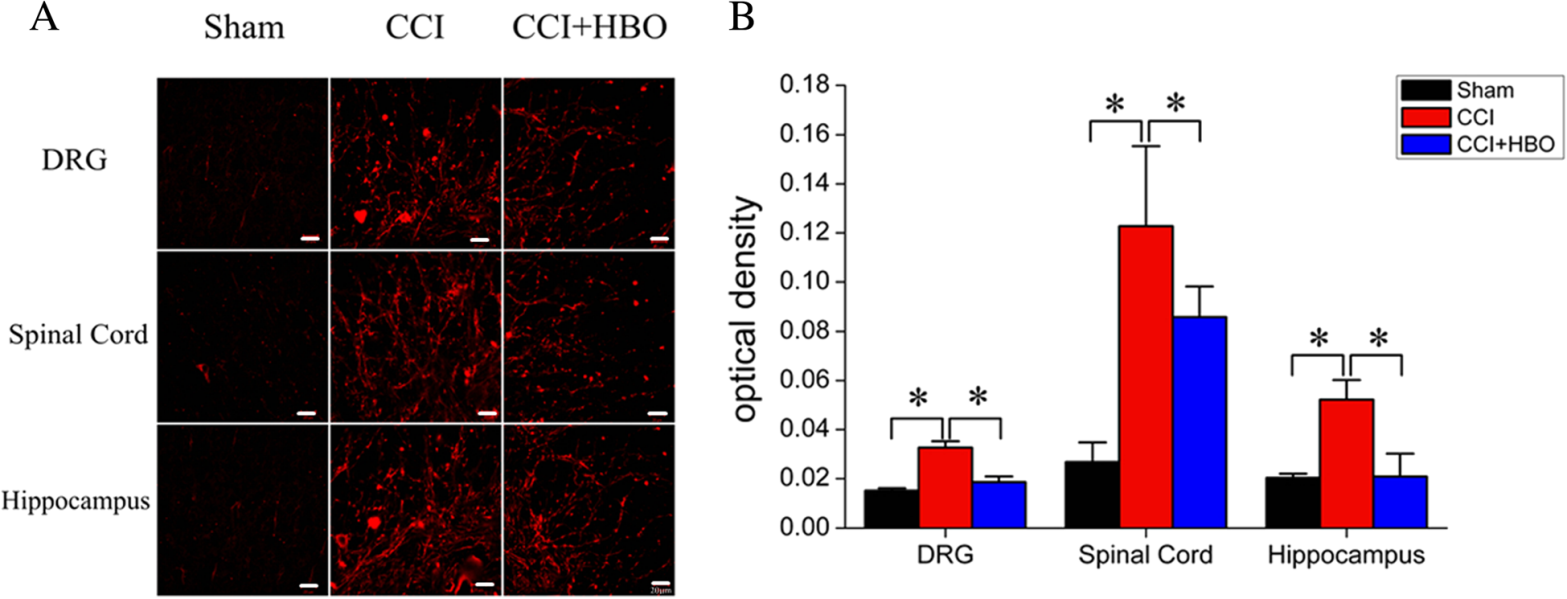
Immunohistochemical analysis of the Kindlin-1 expression in the DRG, spinal cord and hippocampal tissues. **a** On postoperative day 7, tissues were collected and underwent immunohistochemical analysis using an anti-Kindlin-1 antibody. Representative images are presented. **b** The average OD for immunolabeling was calculated from four rats in each group. *P < 0.05

**Fig. 3:**
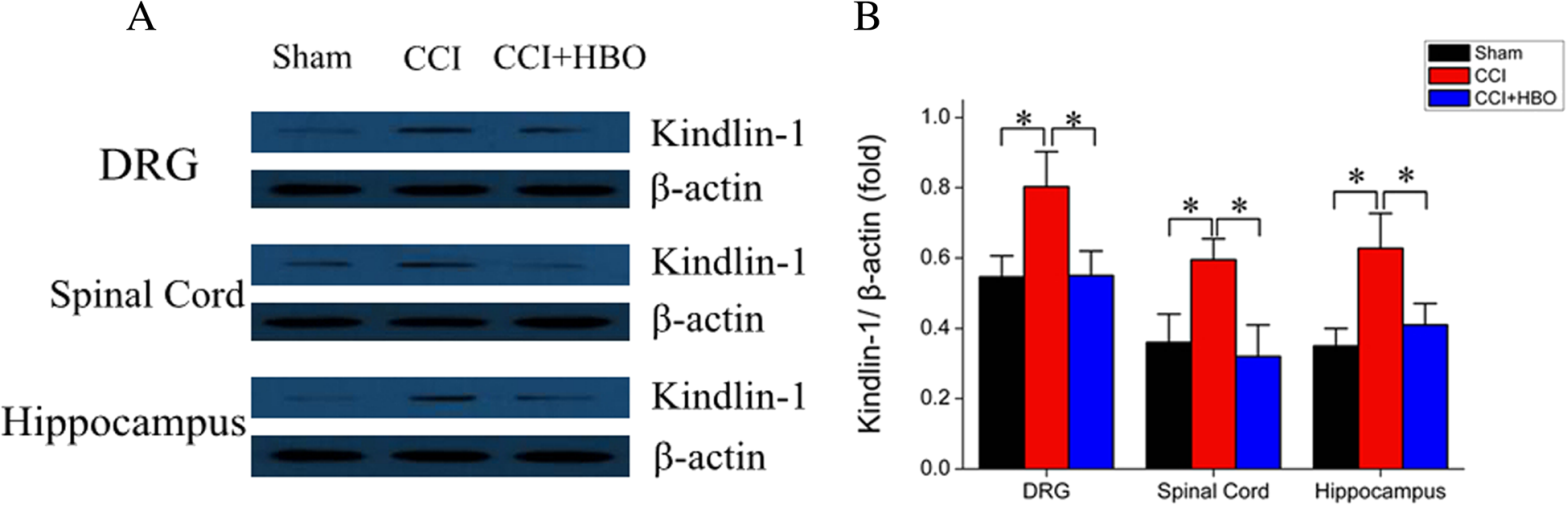
Western blot analysis of Kindlin-1 expression in DRG, spinal cord and hippocampal tissue. **a** On postoperative day 7, tissues were collected and underwent Western blot analysis. Representative images are presented. **b** Kindlin-1 protein expression of was standardized according to β-actin levels. *N* = 4 for each group. *P < 0.05

In order to clarify the molecular mechanisms involved in HBO-mediated analgesia, the expression of Kindlin-1 in the DRG, spinal cord, and hippocampal tissues was determined using immunohistochemistry and Western blot analysis on postoperative day 7. The expression of Kindlin-1 was greatly up-regulated in the DRG, spinal cord, and hippocampal tissues obtained from CCI rats (Fig. 2, *P* < 0.05 *vs.* sham). However, HBO therapy significantly reversed the up-regulation of Kindlin-1 levels induced by CCI (Fig. 3, *P* < 0.05 *vs.* CCI), indicating that Kindlin-1 may play a crucial role in HBO-mediated analgesia in the rat neuropathic pain model.

### HBO suppressed astrocyte activation and TNF-α generation induced by CCI

**Fig. 4:**
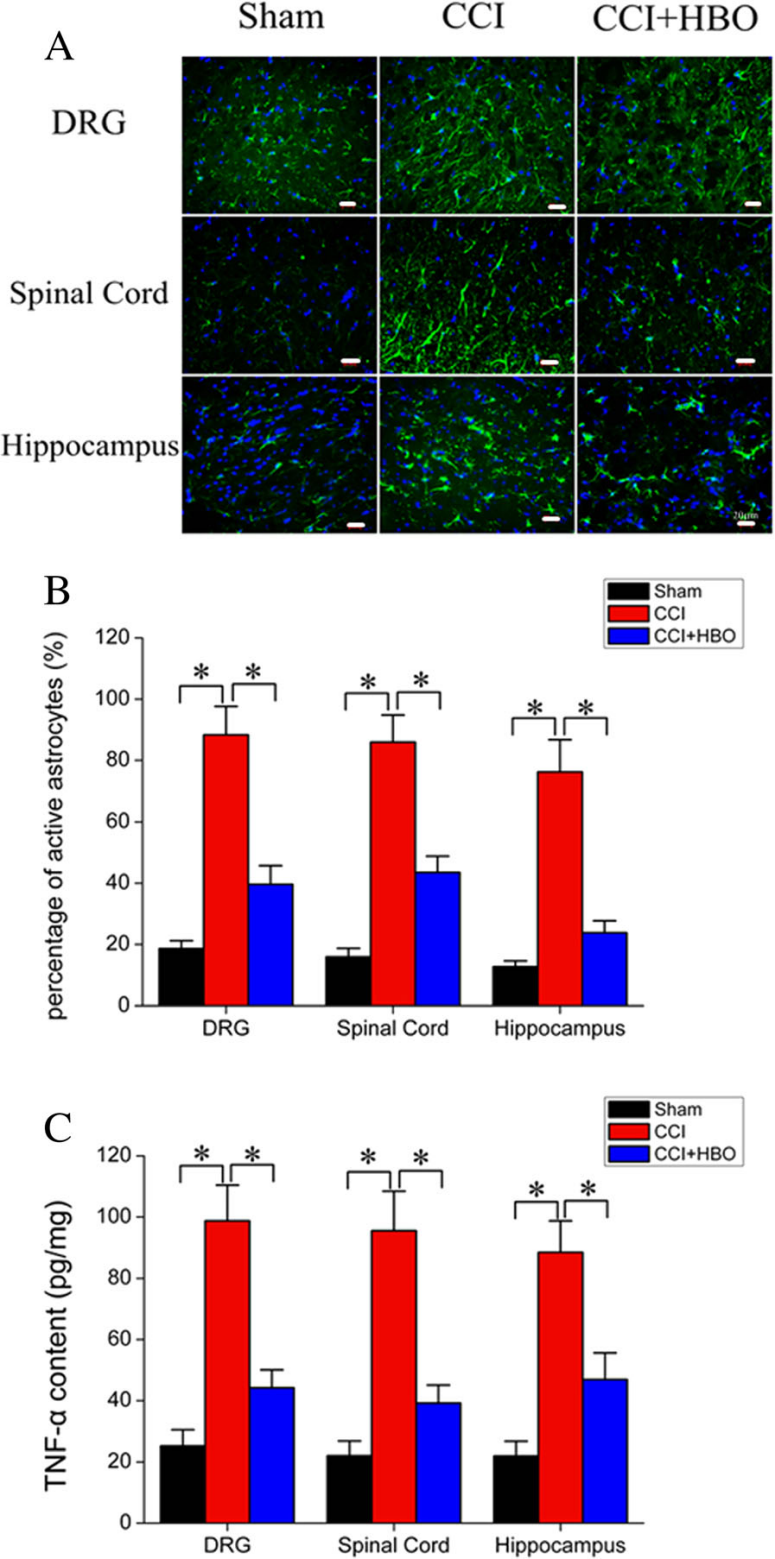
HBO suppresses astrocyte activation. On postoperative day 7, sections of the DRG, spinal cord, and hippocampal tissues were stained with anti-glial fibrillary acidic protein (GFAP) (green) antibody. Nuclei were counterstained with DAPI. **a** Representative micrographs. **b** Quantification of the percentage of GFAP-labeled astrocytes. **c** TNF-α generation was examined by ELISA. *N* = 4 for each group. *P < 0.05

We next determined the potential influence of HBO treatment on astrocyte activation. For this purpose, tissue samples were stained with an anti-GFAP antibody which specifically labels astrocytes. CCI induced the activation of astrocytes in several tissues of the central nervous system, including the DRG, spinal cord, and hippocampus (Fig. 4a, b). HBO therapy significantly reduced astrocyte activation. As astrocyte activation may lead to inflammatory responses through the generation of pro-inflammatory cytokines, such as TNF-α, the production of TNF-α in the aforementioned neuronal tissues was determined using ELISA. As expected, CCI increased TNF-α concentrations when compared with the sham operation control group (*P* < 0.05 *vs.* sham) (Fig. 4c). However, HBO treatment reduced the production of TNF-α following CCI (*P* < 0.05 *vs.* CCI). These data indicate that HBO suppressed astrocyte activation and subsequently prevented astrocyte-induced inflammation.

### HBO reversed Wnt-10 up-regulation induced by CCI

**Fig. 5:**
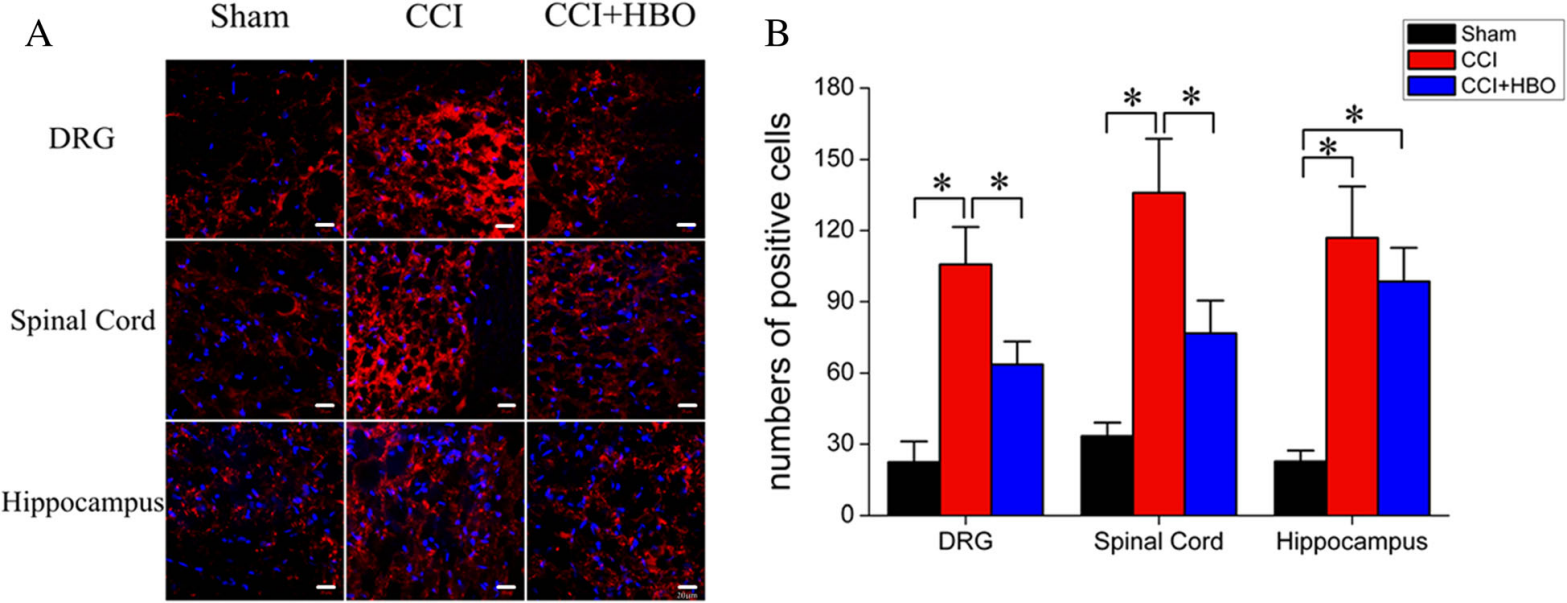
Immunolabeling of Wnt-10a expression in DRG, spinal cord, and hippocampal tissues. **a** On postoperative day 7, tissue samples were immunostained with anti-Wnt-10a antibody. Nuclei were counterstained with DAPI. Representative images were presented. **b** The average number of Wnt-10a-positive cells was calculated from four rats in each group.*P < 0.05

**Fig. 6:**
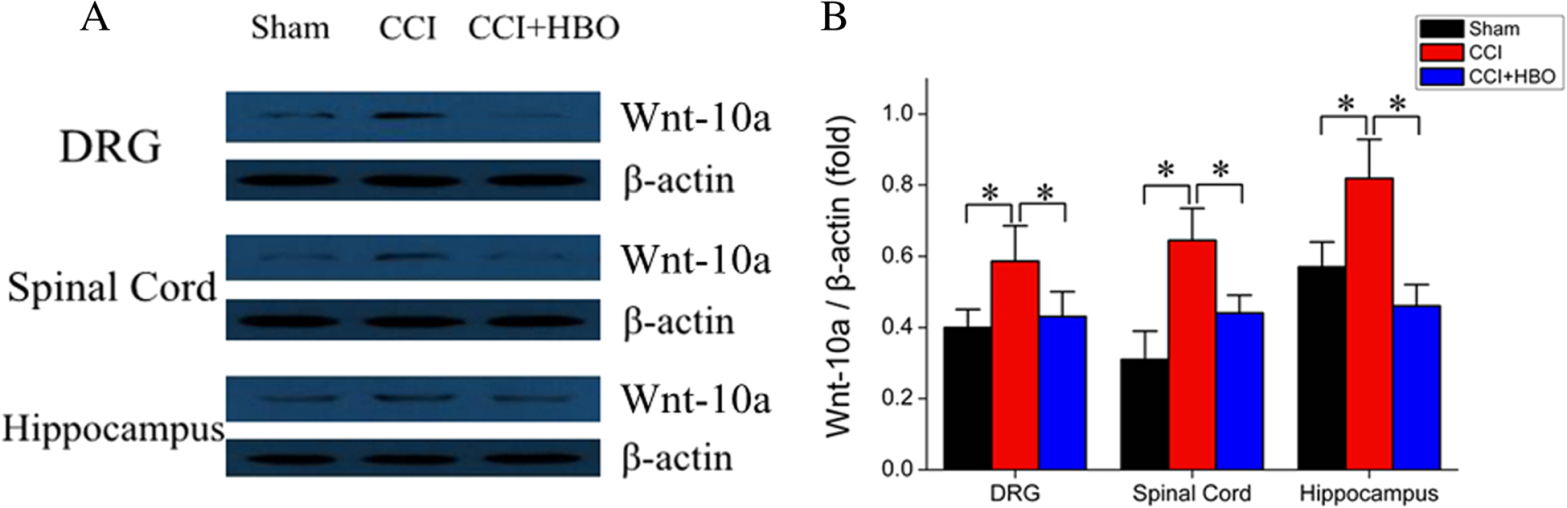
Western blot analysis of Wnt-10a expression in the DRG, spinal cord and hippocampal tissues. **a** On postoperative day 7, tissues were collected and subjected to Western blot analysis. Representative images are presented. **b** Wnt-10a protein expression was standardized according to β-actin levels. *N* = 4 for each group. *P < 0.05

Considering that Wnt signaling lies downstream of Kindlin-1 [17], we next investigated the expression of Wnt-10a in the DRG, spinal cord, and hippocampal regions of animals from different experimental groups. The number of Wnt-10a-positive cells were dramatically increased in the DRG and spinal cord obtained from CCI rats (Fig. 5, *P* < 0.05 *vs.* sham), which was effectively reversed by HBO therapy (*P* < 0.05 *vs.* CCI). Similarly, Western blot analysis further demonstrated that Wnt-10a protein expression was greatly up-regulated in the DRG, spinal cord, and hippocampus of animals in the CCI group (*P* < 0.05 *vs.* sham), whereas HBO treatment significantly attenuated the elevation of Wnt-10a in these neuronal tissues (*P* < 0.05 *vs.* CCI) (Fig. 6). These observations suggest that HBO may reduce neuropathic pain, possibly by regulating the Kindlin-1/Wnt-10a signaling pathway in rat neuronal tissues.

## Discussion

By establishing a rat neuropathic pain model, we were able to investigate the effects of HBO on pain relief after CCI of the sciatic nerve. Our results show that HBO treatment efficiently suppressed mechanical and thermal hypersensitivity in CCI rats. The antinociceptive effects of HBO appear to be related to its action of suppressing astrocyte activation and inflammatory responses via the Kindlin-1/Wnt-10a inflammatory signaling pathway.

HBO treatment has been recognized as a promising non-invasive therapy for a variety of disorders, including neuropathic pain [9]. The antinociceptive efficacy of HBO treatment has been proven in many rodent experiments and also human patients [9–11, 13]. In accordance with these findings, our current study demonstrated that five consecutive days of HBO treatment greatly attenuated neuropathic pain by suppressing hypersensitivity in rats following CCI of the sciatic nerve. Thermal hypersensitivity occurred earlier than mechanical hypersensitivity in rats following CCI, and HBO treatment effectively reduced both hypersensitivities. Based on previous studies and our present findings, HBO therapy may be a novel non-pharmacological approach for alleviating neuropathic pain. However, the efficacy of long-term HBO treatment has yet to be investigated.

Astrocytes, the most abundant cells in the central nervous system, play an essential role in the induction of inflammation and neuropathic pain [20]. Compared with other types of glial cells, such as microglia, significant and persistent activation of astrocytes is a common feature following painful injury [21]. Pro-inflammatory cytokines, such as TNF-α, released from activated astrocyte following injury, contribute to the development of inflammation as well as neuropathic pain [22, 23]. Our previous study showed that on postoperative day 7, there was significant astrocyte activation in the spinal dorsal horn in CCI rats [10], implying a crucial role of spinal cord astrocytes in modulating neuroinflammation in neuropathic pain conditions. In addition to the spinal cord, the DRG participate in the regulation of neuroimmune responses in neuropathic pain, as demonstrated by the differential expression of inflammatory neuropeptides and altered activation of peripheral immune cells in this region after painful injury [24, 25]. The hippocampus, a key integration site for pain signals, regulates CCI-induced pain behavior in rats [26]. Based on this evidence, we focused on investigating astrocyte activation and related inflammatory mechanisms in the DRG, spinal cord, and hippocampus of CCI-treated rats. Our results demonstrated that astrocyte activation occurred and was accompanied by elevated TNF-α levels in central nervous system tissues following CCI. HBO treatment efficiently prevented astrocyte activation and production of TNF-α induced by CCI. Similar results were obtained by other research groups, whereby HBO therapy alleviated CCI-induced neuropathic pain as well as reducing generation of pain [27]. These combined data suggest that HBO therapy may suppress mechanical and thermal hyperalgesia through inhibition of astrocyte activation-evoked neuroinflammation.

A recent study reported that astrocyte activation in response to mechanical and inflammatory stimuli was linked to components of extracellular matrix (ECM) [15]. Inhibition of ECM protein receptors by blockade of β1 integrins suppressed astrocyte responses to ECM components [15]. Kindlin-1 is an integrin binding protein [16], however its effects in astrocyte activation and neuroinflammation in neuropathic pain have not yet been studied. It is conceivable that CCI may induce astrocyte activation through up-regulation of Kindlin-1. A growing body of evidence also suggests that Wnt family members play crucial roles in the pathogenesis of neuropathic pain. Neuronal injury leads to the rapid and persistent activation of Wnt signals, while blockade of the Wnt signaling pathway inhibits the development and progression of neuropathic pain [28–30]. Moreover, Wnt, a downstream effecter of Kindlin-1 [17], has been found to stimulate the release of pro-inflammatory cytokines, such as TNF-α and IL-18, in models of neuropathic pain [28]. In accordance with these observations, increased Wnt-10a expression was detected in the spinal cord, DRG, and hippocampus of CCI-treated rats. Importantly, HBO therapy reversed the up-regulation of Kindlin-1 as well as Wnt-10a caused by CCI, suggesting the antinociceptive effects of HBO treatment may result from Kindlin-1/Wnt signaling-mediated suppression of astrocyte activation and inflammation.

## Conclusions

The role of Kindlin-1/Wnt-10a in pain, astrocyte activation, or neuroinflammation has not yet been clarified. Our present study for the first time, demonstrated that HBO treatment, likely via regulating the Kindlin-1/Wnt-10a signaling pathway, attenuated rat neuropathic pain induced by CCI of rat sciatic nerve. These data suggest that use of HBO may be a novel therapeutic approach in alleviating neuropathic pain in patients. Moreover, interference with the Kindlin-1/Wnt-10a signaling pathway may also prove to be a drug target for reducing neuroinflammatory responses of astrocytes in the pathogenesis of neuropathic pain.

## Abbreviations

CCI: Chronic constriction injury
DRG: Dorsal root ganglia
ECL: Enhanced chemiluminescence
ECM: Extracellular matrix
GFAP: Glial fibrillary acidic protein
HBO: Hyperbaric oxygen
HRP: Horseradish peroxidase
MWT: Mechanical withdrawal threshold
OD: Optical density
PBS: Phosphate buffer solution
SD: Sprague-Dawley
TNF: Tumor necrosis factor
TWL: Thermal withdrawal latency
