# Correction to: Hyperbaric oxygen attenuates neuropathic pain and reverses inflammatory signaling likely via the Kindlin-1/Wnt-10a signaling pathway in the chronic pain injury model in rats

**DOI:** 10.1186/s10194-022-01407-x

**Published:** 2022-03-04

**Authors:** Baisong Zhao, Yongying Pan, Haiping Xu, Xingrong Song

**Affiliations:** Department of Anesthesiology, Guangzhou Women and Children’s Medical Center, Guangzhou Medical University, No. 9 Jinsui Road, Tianhe District, Guangzhou, 510623 Guangdong China

**Correction to: J Headache Pain 18, 1 (2017)**

**https://doi.org/10.1186/s10194-016-0713-y**

Following the publication of the original article [1], we were notified of an error in Fig. 2.

The authors have now provided the correct Fig. 2 (which can be found below) and apologize for the mistake.

**Fig. 2:**
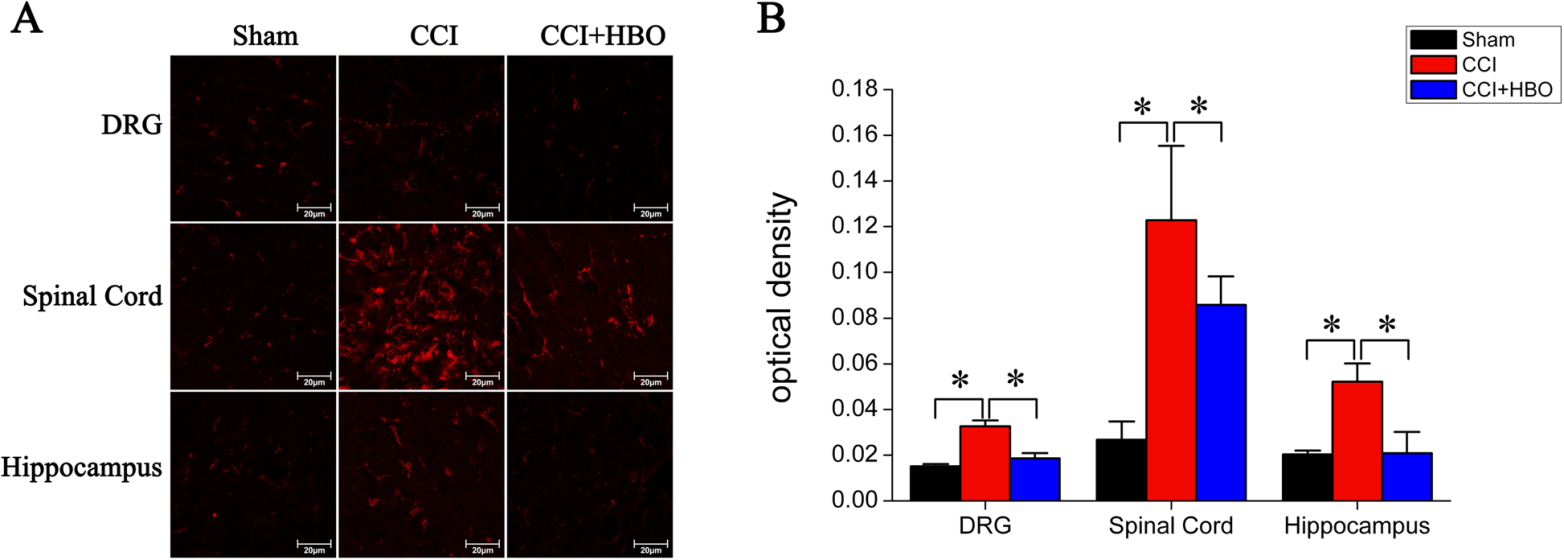
Immunohistochemical analysis of the Kindlin-1 expression in the DRG, spinal cord and hippocampal tissues. **a** On postoperative day 7, tissues were collected and underwent immunohistochemical analysis using an anti-Kindlin-1 antibody. Representative images are presented. **b** The average OD for immunolabeling was calculated from four rats in each group. *P < 0.05
